# Poly[μ-aqua-diaqua­(μ_2_-pyrazine-2,3-dicarboxyl­ato)dilithium(I)]

**DOI:** 10.1107/S1600536809050570

**Published:** 2009-11-28

**Authors:** Mustafa Tombul, Kutalmis Guven

**Affiliations:** a Department of Chemistry, Faculty of Art and Science, University of Kirikkale, Campus, Yahsihan, Kirikkale, 71450 Kirikkale, Turkey; b Department of Physics, Faculty of Art and Science, University of Kirikkale, Campus, Yahsihan, Kirikkale, 71450 Kirikkale, Turkey

## Abstract

The asymmetric unit of the title compound, [Li_2_(C_6_H_2_N_2_O_4_)(H_2_O)_3_]_*n*_, consists of two independent Li^+^ cations, one pyrazine-2,3-dicarboxyl­ate dianion and three water mol­ecules. One of the Li^+^ cations has a distorted tetra­hedral geometry, coordinated by one of the carboxyl­ate O atoms of the pyrazine-2,3-dicarboxyl­ate ligand and three O atoms from three water mol­ecules, whereas the other Li^+^ cation has a distorted trigonal-bipyramidal geometry, coordinated by a carboxyl­ate O atom of a symmetry-related pyrazine-2,3-dicarboxyl­ate ligand, two water mol­ecules and a chelating pyrazine-2,3-dicarboxyl­ate ligand (by utilizing both N and O atoms) of an adjacent mol­ecule. The synthesis of a hydrated polymeric dinuclear lithium complex formed with two pyrazine-2,3-dicarboxylic acid ligands has been reported previously [Tombul *et al.* (2008*a* ▶). *Acta Cryst.* E**64**, m491–m492]. By comparision to the complex reported here, the dinuclear complex formed with two pyrazine-2,3-dicarboxylic acid ligands differs in the coordination geometry of both Li atoms. The crystal structure further features O—H⋯O and O—H⋯N hydrogen-bonding inter­actions involving the water mol­ecules and carboxyl­ate O atoms.

## Related literature

For a general background to multidendate carboxylic acids, see: Erxleben (2003 ▶); Ye *et al.* (2005 ▶); Fei *et al.* (2006 ▶). For further information on pyrazine-2,3-dicarboxylic acid, see: Takusagawa & Shimada (1973 ▶); Richard *et al.* (1973 ▶); Nepveu *et al.* (1993 ▶). For further information on the synthesis of metal complexes with pyrazine-2,3-dicarboxylic acid ligand, see: Tombul & Güven (2009 ▶); Tombul *et al.* (2006 ▶, 2007 ▶, 2008*b* ▶). For a related structure of lithium with pyrazine-2,3-dicarb­oxylic acid ligand, see: Tombul *et al.* (2008*a* ▶). For Li—O bond distances, see: Chen *et al.* (2007 ▶); Kim *et al.* (2007 ▶). For Li—N bond lengths, see: Grossie *et al.* (2006 ▶); Boyd *et al.* (2002 ▶).
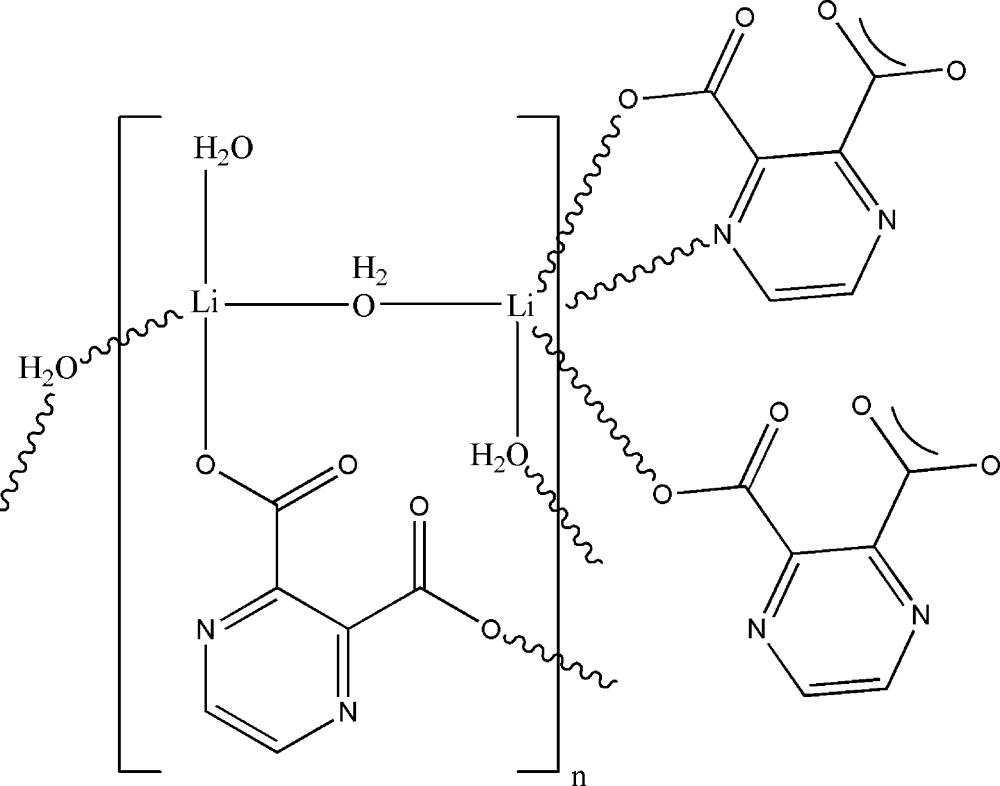


## Experimental

### 

#### Crystal data

[Li_2_(C_6_H_2_N_2_O_4_)(H_2_O)_3_]
*M* *_r_* = 234.02
Monoclinic, 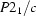

*a* = 7.487 (3) Å
*b* = 16.409 (8) Å
*c* = 7.958 (2) Å
β = 92.92 (3)°
*V* = 976.4 (7) Å^3^
*Z* = 4
Mo *K*α radiation
μ = 0.14 mm^−1^
*T* = 298 K
0.40 × 0.20 × 0.06 mm

#### Data collection

Rigaku AFC-7S diffractometer
Absorption correction: ψ scan (North *et al.*, 1968 ▶) *T*_min_ = 0.948, *T*_max_ = 0.994
6366 measured reflections
6045 independent reflections
2427 reflections with *I* > 2σ(*I*)
*R*_int_ = 0.120
3 standard reflections frequency: 150 reflections intensity decay: none

#### Refinement

*R*[*F* ^2^ > 2σ(*F* ^2^)] = 0.062
*wR*(*F* ^2^) = 0.211
*S* = 0.96
6045 reflections
178 parameters
H atoms treated by a mixture of independent and constrained refinement
Δρ_max_ = 0.49 e Å^−3^
Δρ_min_ = −0.54 e Å^−3^

### 

Data collection: *MSC/AFC Diffractometer Control Software* (Molecular Structure Corporation, 1989 ▶); cell refinement: *MSC/AFC Diffractometer Control Software*; data reduction: *TEXSAN* (Molec­ular Structure Corporation, 1993 ▶); program(s) used to solve structure: *SHELXS97* (Sheldrick, 2008 ▶); program(s) used to refine structure: *SHELXL97* (Sheldrick, 2008 ▶); molecular graphics: *Mercury* (Macrae *et al.*, 2008 ▶); software used to prepare material for publication: *publCIF* (Westrip, 2009 ▶).

## Supplementary Material

Crystal structure: contains datablocks global, I. DOI: 10.1107/S1600536809050570/om2293sup1.cif

Structure factors: contains datablocks I. DOI: 10.1107/S1600536809050570/om2293Isup2.hkl

Additional supplementary materials:  crystallographic information; 3D view; checkCIF report

## Figures and Tables

**Table 1:** Selected bond lengths (Å)

O5—Li1	2.046 (4)
O5—Li2	2.069 (4)
O6—Li2	2.129 (4)
O2—Li1	1.927 (3)
N1—Li2^i^	2.317 (4)
Li2—O1^ii^	1.942 (3)
Li2—O3^iii^	1.988 (3)
Li1—O7	1.918 (4)
Li1—O6^iii^	1.973 (4)

Symmetry codes: (i) 
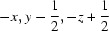
; (ii) 
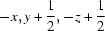
; (iii) 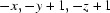.

**Table 2:** Hydrogen-bond geometry (Å, °)

*D*—H⋯*A*	*D*—H	H⋯*A*	*D*⋯*A*	*D*—H⋯*A*
O5—H5*A*⋯O2^iv^	0.81 (3)	1.92 (3)	2.723 (3)	172.60 (3)
O5—H5*B*⋯O4	0.87 (4)	2.28 (4)	3.068 (3)	152 (3)
O6—H6*A*⋯N2^v^	0.86 (3)	2.00 (3)	2.857 (3)	169.66 (4)
O6—H6*B*⋯O3	0.88 (3)	1.86 (3)	2.719 (3)	163 (3)
O7—H7*A*⋯O4^iv^	0.88 (4)	2.02 (4)	2.841 (3)	154.89 (6)
O7—H7*B*⋯O4^vi^	0.88 (4)	1.86 (4)	2.730 (3)	169.51 (6)

Symmetry codes: (iv) 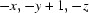; (v) 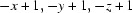; (vi) 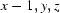.

**Fig. 1.**
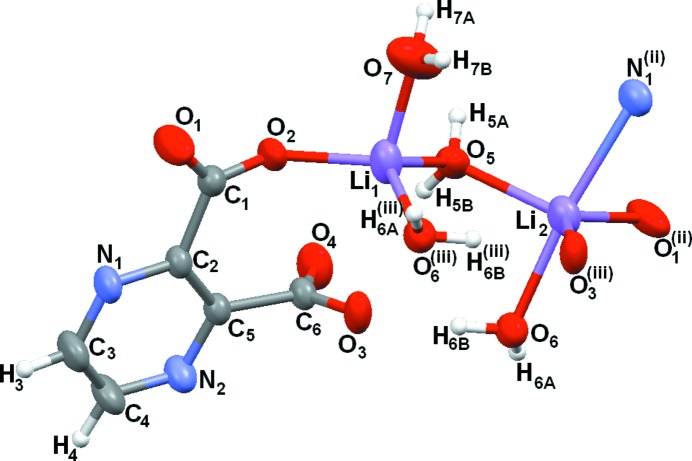
Showing the atom-labelling scheme with symmetry codes: ii = -x, y + 1/2, -z + 1/2; iii = -x, -y + 1, -z + 1.)] Atoms were drawn with 50% thermal ellipsoid probability contours.

**Fig. 2.**
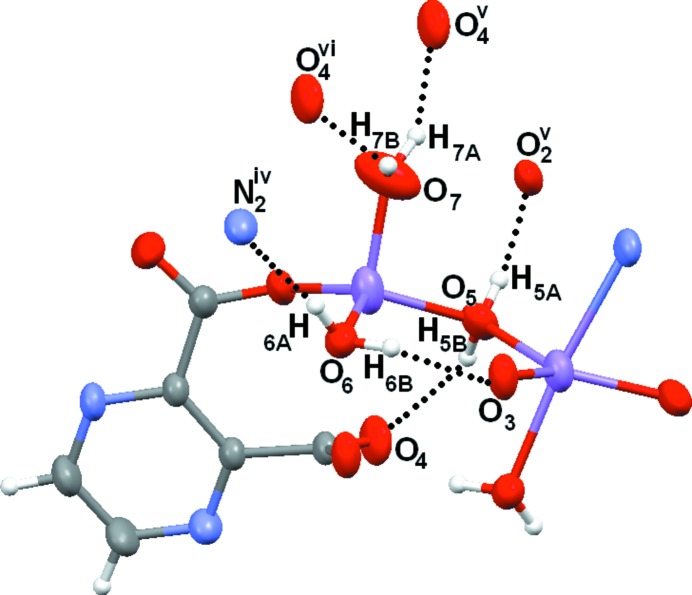
A view of the hydrogen bonding interactions in the structure. Symmetry codes: iv = -x + 1, -y + 1, -z + 1; v = -x, -y + 1, -z; vi = x - 1, y, z]. Atoms were drawn with 50% thermal ellipsoid probability contours.

## supplementary crystallographic information

### Comment

Multidendate carboxylic acids are found to be excellent ligands for the synthesis of coordination polymers, giving structures with a diverse range of topologies and conformations, owing to the carboxylate groups being able to coordinate to a metal centre as a mono-, bi-, or multidentate ligand (Erxleben, 2003; Ye *et al.*, 2005; Fei *et al.*, 2006). Pyrazine-2,3-dicarboxylic acid (Takusagawa & Shimada, 1973) and its dianion (Richard *et al.*, 1973; Nepveu *et al.*, 1993) have been reported to be well suited for the construction of multidimentional frameworks (nD, n = 1–3), due to the presence of two adjacent carboxylate groups (O donor atoms) as substituents on the N-heterocyclic pyrazine ring (N donor atoms). In recent years, metal complexes with pyrazine-2,3-dicarboxylic acid ligand have been extensively studied because of their wide applications and growing interest in supramolecular chemistry. Examples include sodium (Tombul *et al.*, 2006), caesium (Tombul *et al.*, 2007), potassium (Tombul *et al.*, 2008*b*), lithium (Tombul *et al.*, 2008*a*) and rubidium (Tombul & Guven, 2009) complexes. As a continuation of our ongoing research on Group I dicarboxylates, we report here the synthesis and crystal structure of the hydrated polymeric dinuclear lithium complex formed with one molar equivalent of pyrazine-2,3-dicarboxylic acid.

As shown in Fig. 1, the title compound is a polymeric dinuclear complex with two kinds of Li atoms, one pyrazine-2,3-dicarboxylate ligand and three water molecules in the asymmetric unit. The geometries of the two independent Li atoms are different and the coordination modes of the pyrazine-2,3-dicarboxylate towards the cations are dissimilar. The Li1 ion has a distorted four-coordinate geometry and achieves the coordination number by bonding to one of the carboxylate O atom of pyrazine-2,3-dicarboxylate ligand, three O atoms from three water molecules, one of which is a symmetry-related bridging O atom. The Li2 ion has a distorted trigonal bipyramidal geometry, with one water molecule in bridging mode that connects the two distinct Li ions, one symmetry related carboxylate O atom of pyrazine-2,3-dicarboxylate ligand and a chelated pyrazine-2,3-dicarboxylate ligand (through the interactions of both N and O atoms) of the adjacent molecule. It should be emphasized that, depending on the starting material and stoichiometric ratio utilized, the synthesis of dinuclear lithium complexes formed with one or two pyrazine-2,3-dicarboxylic acid ligands can be accessible (Tombul *et al.*, 2008a). The Li–O distances are in the range 1.918 (4)Å to 2.046 (4)Å (for Li1) and 1.942 (3)Å to 2.129 (4)Å (for Li2), in good agreement with the corresponding values reported for other lithium complexes (Chen *et al.*, 2007; Kim *et al.*, 2007). It is interesting to note that Li–N bond lengths are in accord with the normal ranges reported for the dinuclear bis-structure (Tombul *et al.*, 2008*a*), however, the Li–N distances are notably longer than similar bond lengths reported in the literature (Grossie *et al.*, 2006; Boyd *et al.*, 2002). The dinuclear complex is linked in a three-dimensional manner by further intra- and intermolecular O—H–O and O—H–N hydrogen bonds (Figure 2 and Table 2).

### Experimental

To an aqueous solution (30 ml) of pyrazine 2,3-dicarboxylic acid (1681 mg, 1 mmol), LiOH (479 mg, 2 mmol) was carefully added. The reaction mixture gave a colourless and clear solution which was stirred at 303 K for 4 h. After solvent removal in vacuo, the white solid product was then redissolved in water (5 ml) and allowed to stand for 15 d at ambient temperature, after which transparent fine crystals were harvested from the mother liquor.

### Refinement

H atoms associated with water molecules were located in the difference map and freely refined during subsequent cycles of least squares. H atoms of carbons were repositioned geometrically. They were initially refined with soft restraints on the bond lengths and angles to regularize their geometry (C—H = 0.93 Å) and Uĩso~(H) (in the range 1.2–1.5 times U~eq~ of the parent atom) ,after which the positions were refined with riding constraints.

### Crystal data

**Table:**

[Li_2_(C_6_H_2_N_2_O_4_)(H_2_O)_3_]	*F*(000) = 480
*M**_r_* = 234.02	*D*_x_ = 1.592 Mg m^−^^3^
Monoclinic, *P*2_1_/*c*	Mo *K*α radiation, λ = 0.71069 Å
Hall symbol: -P 2ybc	Cell parameters from 25 reflections
*a* = 7.487 (3) Å	θ = 3.0–7.9°
*b* = 16.409 (8) Å	µ = 0.14 mm^−^^1^
*c* = 7.958 (2) Å	*T* = 298 K
β = 92.92 (3)°	Prism, yellow
*V* = 976.4 (7) Å^3^	0.4 × 0.2 × 0.06 mm
*Z* = 4	

### Data collection

**Table:**

Rigaku diffractometer	2427 reflections with *I* > 2σ(*I*)
Radiation source: fine-focus sealed tube	*R*_int_ = 0.120
graphite	θ_max_ = 40.0°, θ_min_ = 2.7°
ω–2θ scans	*h* = 0→13
Absorption correction: ψ scan (North *et al.*, 1968)	*k* = 0→29
*T*_min_ = 0.948, *T*_max_ = 0.994	*l* = −14→14
6366 measured reflections	3 standard reflections every 150 reflections
6045 independent reflections	intensity decay: none

### Refinement

**Table:**

Refinement on *F*^2^	Primary atom site location: structure-invariant direct methods
Least-squares matrix: full	Secondary atom site location: difference Fourier map
*R*[*F*^2^ > 2σ(*F*^2^)] = 0.062	Hydrogen site location: inferred from neighbouring sites
*wR*(*F*^2^) = 0.211	H atoms treated by a mixture of independent and constrained refinement
*S* = 0.96	*w* = 1/[σ^2^(*F*_o_^2^) + (0.0977*P*)^2^] where *P* = (*F*_o_^2^ + 2*F*_c_^2^)/3
6045 reflections	(Δ/σ)_max_ < 0.001
178 parameters	Δρ_max_ = 0.49 e Å^−^^3^
0 restraints	Δρ_min_ = −0.54 e Å^−^^3^

### Special details

**Table:**

Geometry. All e.s.d.'s (except the e.s.d. in the dihedral angle between two l.s. planes) are estimated using the full covariance matrix. The cell e.s.d.'s are taken into account individually in the estimation of e.s.d.'s in distances, angles and torsion angles; correlations between e.s.d.'s in cell parameters are only used when they are defined by crystal symmetry. An approximate (isotropic) treatment of cell e.s.d.'s is used for estimating e.s.d.'s involving l.s. planes.
Refinement. Refinement of *F*^2^ against ALL reflections. The weighted *R*-factor *wR* and goodness of fit *S* are based on *F*^2^, conventional *R*-factors *R* are based on *F*, with *F* set to zero for negative *F*^2^. The threshold expression of *F*^2^ > σ(*F*^2^) is used only for calculating *R*-factors(gt) *etc*. and is not relevant to the choice of reflections for refinement. *R*-factors based on *F*^2^ are statistically about twice as large as those based on *F*, and *R*- factors based on ALL data will be even larger.

### Fractional atomic coordinates and isotropic or equivalent isotropic displacement parameters (Å^2^)

**Table:**

	*x*	*y*	*z*	*U*_iso_*/*U*_eq_	
O1	−0.01123 (18)	0.24672 (8)	0.0505 (2)	0.0341 (3)	
O2	0.06063 (17)	0.37715 (7)	0.09640 (16)	0.0236 (3)	
O3	0.20616 (17)	0.43281 (7)	0.43623 (16)	0.0238 (3)	
O4	0.39167 (18)	0.47554 (7)	0.24601 (18)	0.0277 (3)	
O5	0.03262 (19)	0.56609 (8)	0.21735 (17)	0.0252 (3)	
H5A	0.004 (4)	0.5875 (17)	0.129 (4)	0.043 (8)*	
H5B	0.147 (5)	0.558 (2)	0.222 (4)	0.069 (10)*	
O6	0.17467 (17)	0.58515 (8)	0.56590 (16)	0.0224 (2)	
H6A	0.272 (4)	0.613 (2)	0.581 (4)	0.058 (9)*	
H6B	0.206 (4)	0.5391 (19)	0.519 (3)	0.048 (8)*	
O7	−0.2951 (2)	0.46974 (13)	0.0850 (2)	0.0479 (5)	
H7A	−0.316 (5)	0.501 (2)	−0.004 (5)	0.082 (12)*	
H7B	−0.394 (5)	0.465 (2)	0.139 (4)	0.064 (10)*	
N1	0.29092 (19)	0.19673 (8)	0.21499 (19)	0.0228 (3)	
N2	0.52735 (19)	0.30911 (9)	0.3651 (2)	0.0246 (3)	
C1	0.0838 (2)	0.30153 (9)	0.1146 (2)	0.0195 (3)	
C2	0.2524 (2)	0.27631 (9)	0.2158 (2)	0.0172 (3)	
C3	0.4472 (3)	0.17365 (11)	0.2881 (3)	0.0296 (4)	
H3	0.4761	0.1185	0.2920	0.036*	
C4	0.5669 (2)	0.22967 (11)	0.3582 (3)	0.0300 (4)	
H4	0.6777	0.2118	0.4017	0.036*	
C5	0.3672 (2)	0.33240 (9)	0.2984 (2)	0.0171 (3)	
C6	0.31766 (19)	0.42055 (9)	0.3264 (2)	0.0168 (3)	
Li1	−0.0795 (5)	0.4524 (2)	0.2209 (4)	0.0289 (7)	
Li2	−0.0490 (4)	0.63229 (18)	0.4210 (4)	0.0252 (6)	

### Atomic displacement parameters (Å^2^)

**Table:**

	*U*^11^	*U*^22^	*U*^33^	*U*^12^	*U*^13^	*U*^23^
O1	0.0312 (7)	0.0205 (6)	0.0488 (9)	−0.0017 (5)	−0.0159 (6)	−0.0045 (6)
O2	0.0304 (6)	0.0161 (5)	0.0240 (6)	0.0064 (4)	−0.0028 (5)	−0.0005 (4)
O3	0.0310 (6)	0.0158 (5)	0.0257 (6)	0.0022 (4)	0.0123 (5)	0.0002 (4)
O4	0.0300 (6)	0.0176 (5)	0.0367 (7)	−0.0035 (4)	0.0129 (5)	0.0050 (5)
O5	0.0298 (6)	0.0255 (6)	0.0201 (6)	0.0068 (5)	0.0016 (5)	0.0034 (5)
O6	0.0224 (5)	0.0192 (5)	0.0253 (6)	−0.0002 (4)	−0.0005 (4)	−0.0020 (4)
O7	0.0300 (8)	0.0775 (14)	0.0371 (9)	0.0162 (8)	0.0109 (6)	0.0208 (9)
N1	0.0256 (7)	0.0129 (5)	0.0297 (7)	0.0026 (5)	−0.0004 (5)	−0.0022 (5)
N2	0.0191 (6)	0.0208 (6)	0.0334 (8)	0.0025 (5)	−0.0024 (5)	−0.0025 (5)
C1	0.0225 (7)	0.0161 (6)	0.0197 (7)	0.0025 (5)	−0.0008 (5)	−0.0017 (5)
C2	0.0178 (6)	0.0133 (5)	0.0209 (7)	0.0012 (5)	0.0024 (5)	−0.0005 (5)
C3	0.0296 (8)	0.0169 (7)	0.0417 (11)	0.0074 (6)	−0.0043 (7)	−0.0015 (7)
C4	0.0229 (7)	0.0224 (7)	0.0438 (11)	0.0083 (6)	−0.0060 (7)	−0.0020 (7)
C5	0.0180 (6)	0.0132 (5)	0.0201 (7)	0.0007 (5)	0.0022 (5)	0.0000 (5)
C6	0.0174 (6)	0.0120 (5)	0.0212 (7)	−0.0002 (4)	0.0016 (5)	0.0002 (5)
Li1	0.0367 (17)	0.0197 (13)	0.0312 (17)	0.0031 (12)	0.0100 (13)	0.0010 (12)
Li2	0.0300 (15)	0.0163 (12)	0.0294 (16)	0.0004 (11)	0.0045 (12)	0.0027 (11)

### Geometric parameters (Å, °)

**Table:**

O5—Li1	2.046 (4)	N2—C4	1.338 (2)
O5—Li2	2.069 (4)	N2—C5	1.342 (2)
O5—H5B	0.87 (4)	C2—C5	1.400 (2)
O5—H5A	0.80 (3)	C2—C1	1.519 (2)
O4—C6	1.2517 (19)	C5—C6	1.513 (2)
O6—Li2	2.129 (4)	C4—C3	1.382 (3)
O6—H6A	0.87 (3)	C4—H4	0.9300
O6—H6B	0.88 (3)	C3—H3	0.9300
O1—C1	1.241 (2)	Li2—O1^ii^	1.942 (3)
O2—C1	1.260 (2)	Li2—O3^iii^	1.988 (3)
O2—Li1	1.927 (3)	Li2—N1^ii^	2.317 (4)
O3—C6	1.2552 (19)	Li1—O7	1.918 (4)
N1—C3	1.335 (2)	Li1—O6^iii^	1.973 (4)
N1—C2	1.337 (2)	O7—H7A	0.88 (4)
N1—Li2^i^	2.317 (4)	O7—H7B	0.88 (4)
			
Li1—O5—Li2	109.27 (14)	N1—C3—C4	121.58 (16)
Li1—O5—H5B	105 (2)	N1—C3—H3	119.2
Li2—O5—H5B	112 (2)	C4—C3—H3	119.2
Li1—O5—H5A	109 (2)	O1—C1—O2	126.41 (15)
Li2—O5—H5A	112 (2)	O1—C1—C2	117.66 (14)
H5B—O5—H5A	109 (3)	O2—C1—C2	115.79 (14)
Li1^iii^—O6—Li2	105.71 (15)	O4—C6—O3	124.60 (14)
Li1^iii^—O6—H6A	112 (2)	O4—C6—C5	119.72 (14)
Li2—O6—H6A	121 (2)	O3—C6—C5	115.64 (13)
Li1^iii^—O6—H6B	102.5 (18)	O1^ii^—Li2—O3^iii^	126.42 (18)
Li2—O6—H6B	107.5 (18)	O1^ii^—Li2—O5	121.53 (17)
H6A—O6—H6B	106 (3)	O3^iii^—Li2—O5	111.87 (15)
C1—O1—Li2^i^	121.88 (15)	O1^ii^—Li2—O6	96.70 (15)
C1—O2—Li1	130.35 (15)	O3^iii^—Li2—O6	88.14 (13)
C6—O3—Li2^iii^	138.22 (14)	O5—Li2—O6	88.74 (13)
C3—N1—C2	117.34 (14)	O1^ii^—Li2—N1^ii^	77.56 (12)
C3—N1—Li2^i^	136.14 (14)	O3^iii^—Li2—N1^ii^	92.34 (14)
C2—N1—Li2^i^	106.51 (13)	O5—Li2—N1^ii^	97.38 (14)
C4—N2—C5	117.17 (15)	O6—Li2—N1^ii^	173.18 (17)
N1—C2—C5	121.12 (14)	O7—Li1—O2	105.65 (18)
N1—C2—C1	115.90 (13)	O7—Li1—O6^iii^	101.58 (17)
C5—C2—C1	122.89 (13)	O2—Li1—O6^iii^	118.14 (18)
N2—C5—C2	120.88 (14)	O7—Li1—O5	101.05 (17)
N2—C5—C6	115.78 (13)	O2—Li1—O5	110.05 (17)
C2—C5—C6	123.27 (13)	O6^iii^—Li1—O5	117.60 (17)
N2—C4—C3	121.58 (16)	Li1—O7—H7A	130 (3)
N2—C4—H4	119.2	Li1—O7—H7B	115 (2)
C3—C4—H4	119.2	H7A—O7—H7B	109 (3)
			
C3—N1—C2—C5	−3.5 (2)	C5—C2—C1—O2	6.1 (2)
Li2^i^—N1—C2—C5	176.03 (15)	Li2^iii^—O3—C6—O4	174.97 (19)
C3—N1—C2—C1	173.33 (16)	Li2^iii^—O3—C6—C5	−7.5 (3)
Li2^i^—N1—C2—C1	−7.18 (18)	N2—C5—C6—O4	73.8 (2)
C4—N2—C5—C2	−4.0 (3)	C2—C5—C6—O4	−109.25 (19)
C4—N2—C5—C6	172.98 (16)	N2—C5—C6—O3	−103.86 (18)
N1—C2—C5—N2	6.5 (3)	C2—C5—C6—O3	73.1 (2)
C1—C2—C5—N2	−170.03 (15)	Li1—O5—Li2—O1^ii^	−174.09 (18)
N1—C2—C5—C6	−170.25 (15)	Li1—O5—Li2—O3^iii^	1.3 (2)
C1—C2—C5—C6	13.2 (2)	Li1—O5—Li2—O6	88.83 (16)
C5—N2—C4—C3	−1.1 (3)	Li1—O5—Li2—N1^ii^	−94.19 (16)
C2—N1—C3—C4	−1.6 (3)	Li1^iii^—O6—Li2—O1^ii^	105.93 (16)
Li2^i^—N1—C3—C4	179.05 (19)	Li1^iii^—O6—Li2—O3^iii^	−20.53 (16)
N2—C4—C3—N1	4.1 (3)	Li1^iii^—O6—Li2—O5	−132.46 (14)
Li2^i^—O1—C1—O2	176.24 (17)	C1—O2—Li1—O7	−101.2 (2)
Li2^i^—O1—C1—C2	0.7 (2)	C1—O2—Li1—O6^iii^	11.5 (3)
Li1—O2—C1—O1	87.8 (3)	C1—O2—Li1—O5	150.45 (16)
Li1—O2—C1—C2	−96.7 (2)	Li2—O5—Li1—O7	91.67 (18)
N1—C2—C1—O1	5.3 (2)	Li2—O5—Li1—O2	−156.99 (16)
C5—C2—C1—O1	−177.97 (16)	Li2—O5—Li1—O6^iii^	−17.8 (2)
N1—C2—C1—O2	−170.67 (15)		

Symmetry codes: (i) −*x*, *y*−1/2, −*z*+1/2; (ii) −*x*, *y*+1/2, −*z*+1/2; (iii) −*x*, −*y*+1, −*z*+1.

### Hydrogen-bond geometry (Å, °)

**Table:**

*D*—H···*A*	*D*—H	H···*A*	*D*···*A*	*D*—H···*A*
O5—H5A···O2^iv^	0.81 (3)	1.92 (3)	2.723 (3)	172.60 (3)
O5—H5B···O4	0.87 (4)	2.28 (4)	3.068 (3)	152 (3)
O6—H6A···N2^v^	0.86 (3)	2.00 (3)	2.857 (3)	169.66 (4)
O6—H6B···O3	0.88 (3)	1.86 (3)	2.719 (3)	163 (3)
O7—H7A···O4^iv^	0.88 (4)	2.02 (4)	2.841 (3)	154.89 (6)
O7—H7B···O4^vi^	0.88 (4)	1.86 (4)	2.730 (3)	169.51 (6)

Symmetry codes: (iv) −*x*, −*y*+1, −*z*; (v) −*x*+1, −*y*+1, −*z*+1; (vi) *x*−1, *y*, *z*.
